# [^18^F]FDG PET/CT in head and neck squamous cell carcinoma: a head-to-head between visual point-scales and the added value of multi-modality imaging

**DOI:** 10.1186/s12880-023-00989-5

**Published:** 2023-02-22

**Authors:** Cristina Ferrari, Giulia Santo, Paolo Mammucci, Dino Rubini, Alessio Sciacqua, Angela Sardaro, Antonio Rosario Pisani, Giuseppe Rubini

**Affiliations:** 1 Nuclear Medicine Unit, Interdisciplinary Department of Medicine, University of Bari Aldo Moro, Piazza Giulio Cesare 11, 70124 Bari, Italy; 2 Section of Radiology and Radiation Oncology, Interdisciplinary Department of Medicine, University of Bari Aldo Moro, Piazza Giulio Cesare 11, 70124 Bari, Italy

**Keywords:** HNSCC, PET/CT, Hopkins, Cuneo, Visual score

## Abstract

**Background:**

Head and neck squamous cell carcinoma (HNSCC) represents the 6th leading cancer worldwide. In most cases, patients present a locally advanced disease at diagnosis and non-surgical curative treatment is considered the standard of care. Nowadays, [^18^F]FDG PET/CT is a validated tool in post-treatment evaluation, with a high level of evidence. However, to standardize imaging response, several visual scales have been proposed with none of them approved yet. The study’s aim is a head-to-head comparison between the diagnostic performance of the Hopkins criteria, the Deauville score, and the new proposed Cuneo score, to establish their prognostic role. Secondly, we investigate the possible value of semiquantitative analysis, evaluating SUV_max_ and ΔSUV_max_ of the lymph node with the highest uptake on the restaging PET scan. Moreover, we also considered morphological features using the product of diameters measured on the co-registered CT images to assess the added value of hybrid imaging.

**Methods:**

We performed a retrospective analysis on histologically proven HNSCC patients who underwent baseline and response assessment [^18^F]FDG PET/CT. Post-treatment scans were reviewed according to Hopkins, Deauville, and Cuneo criteria, assigning a score to the primary tumor site and lymph nodes. A per-patient final score for each scale was chosen, corresponding to the highest score between the two sites. Diagnostic performance was then calculated for each score considering any evidence of locoregional progression in the first 3 months as the gold standard. Survival analysis was performed using the Kaplan–Meier method. SUV_max_ and its delta, as well as the product of diameters of the lymph node with the highest uptake at post-treatment scan, if present, were calculated.

**Results:**

A total of 43 patients were finally included in the study. Sensitivity, specificity, PPV, NPV, and accuracy were 87%, 86%, 76%, 92%, and 86% for the Hopkins score, whereas 93%, 79%, 70%, 96%, and 84% for the Deauville score, respectively. Conversely, the Cuneo score reached the highest specificity and PPV (93% and 78%, respectively) but the lowest sensitivity (47%), NPV (76%), and accuracy (77%). Each scale significantly correlated with PFS and OS. The ROC analysis of the combination of SUV_max_ and the product of diameters of the highest lymph node on the restaging PET scan reached an AUC of 0.822. The multivariate analysis revealed the Cuneo criteria and the product of diameters as prognostic factors for PFS.

**Conclusions:**

Each visual score statistically correlated with prognosis thus demonstrating the reliability of point-scale criteria in HNSCC. The novel Cuneo score showed the highest specificity, but the lowest sensibility compared to Hopkins and Deauville criteria. Furthermore, the combination of PET data with morphological features could support the evaluation of equivocal cases.

## Background

Head and Neck Squamous Cell Carcinoma (HNSCC) represents the 6th leading cancer worldwide [1]. After the primary treatment, the recurrence rate within 2 years accounts for approximately 60% of patients, with 20–30% experiencing distant metastases, underlining the need for reliable post-treatment evaluation and proper follow-up [2].

In most cases, patients present a locally advanced disease at diagnosis (stage III or IV) with nodal involvement. In this setting, the combined chemo-radiotherapy (CRT) approach or, less frequently, radiotherapy alone (RT) with curative intent is considered the standard of care. After the end of therapy, a clinical assessment is needed to guide the following decision-making [3].

It is well known that treatment-related changes, particularly edema and fibrosis after high radiation dose, could often limit morphological imaging in post-treatment evaluation [4–6]. Nowadays, fluorine-18 fluorodeoxyglucose positron emission tomography/computed tomography ([^18^F]FDG PET/CT) is a validated tool in post-treatment HNSCC evaluation, with a high level of evidence. The most recent guidelines recommend the use of [^18^F]FDG PET/CT preferably 12 weeks after the end of treatment, pointing out a PET-guided approach. In case of a negative PET exam, the patient is followed-up with clinical observation; in doubtful cases, the patient can be subjected to clinical observation or can repeat the [^18^F]FDG PET/CT in 3–6 months. Finally, a clear PET positivity requests biopsy, primary surgery, or lymphadenectomy after CT or MRI confirmation [3].

In 2016, the PET-NECK, randomized, controlled trial data demonstrated that [^18^F]FDG PET/CT is an accurate and cost-effective technique for HNSCC response assessment, sparing unnecessary neck dissection in nearly 80% of patients [7]. Notably, the metabolic assessment showed a high negative predictive value (NPV) assessing its essential role in HNSCC surveillance [8, 9].

Over the last few years, the need to clarify the diagnostic value of [^18^F]FDG PET/CT even in doubtful and positive cases has promptly emerged. In order to better distinguish the disease from treatment-related changes, some authors have evaluated the prognostic role of several semiquantitative parameters, such as maximum standardized uptake value (SUV_max_), but failed to find out a reliable method [4, 10, 11].

Therefore, there has been growing interest in optimizing the qualitative analysis by proposing different visual scales to standardize the metabolic imaging response. The first scoring system, named the Hopkins Criteria (HC), was introduced by Marcus and colleagues in a retrospective study considering a cohort of 214 patients. The 5-point scale achieved an NPV and overall diagnostic accuracy of 91.1% and 86.9%, respectively. These results were confirmed by the prospective multicenter ECLYPS study, where the use of HC resulted in a NPV of 92.1% [12, 13]. However, considering the studies’ bias as well as the low positive predictive value (PPV), other PET qualitative scores have recently been proposed: the well-known Deauville score (DS) and the novel Cuneo score (CS). In particular, CS, a 6-point visual scale, was introduced to minimize false-negative and false-positive results, resulting in a higher PPV to better discriminate persistent disease from treatment-related changes (e.g. inflammatory reactions after RT) [14]. In addition, each scale takes into account different visual reference regions (e.g. internal jugular vein (IJV); mediastinal blood pool (MBP); liver); only the CS introduces the absence/presence of the local background in PET images evaluation to highlight the possible interference of the post-treatment reaction of local tissue that impacts on visual score interpretation.

The first goal of this study was a head-to-head comparison between the diagnostic performance of the Hopkins criteria, Deauville score, and Cuneo score, together with the assessment of their prognostic role.

Secondly, we extracted SUV_max_ and ΔSUV_max_ of the lymph node with the highest uptake and we calculated the product of diameters measured on the co-registered CT images on the restaging PET/CT scan to investigate the possible added value of semiquantitative analysis and morphological data as a useful variable to consider in doubtful cases.

## Methods

### Patients’ selection

Data about patients with histologically proven non-metastatic locally advanced HNSCC, who were referred to our institution between February 2011 and January 2021, were retrospectively reviewed. All patients eligible for non-surgical curative-intent treatment (radiotherapy alone or chemoradiotherapy), who underwent both baseline and response assessment [^18^F]FDG PET/CT were included for analysis. Exclusion criteria included: previous resection of primary or nodal disease; prior radiotherapy to the head and neck district; other primary cancers. Response assessment PET/CT was performed at least 12 weeks after the end of treatment. All demographics, baseline characteristics, staging, treatment, and outcome details were recorded. Human papillomavirus (HPV) status was also collected, if available.

### [^18^F]FDG PET/CT examination and analysis

All PET/CT scans included in the study were performed according to our institute’s clinical scanning protocols. Acquisitions were performed using a Discovery 710 PET/CT scanner (GE, General Electrics, Milwaukee, WI, USA). The field of view and pixel size of the PET images reconstructed for fusion was 70 cm and 2.73 mm respectively, with a matrix size of 256 × 256. The technical parameters used for CT imaging were: pitch 0.98, gantry rotation speed of 0.5 s/rot, 120 kVp, and modulated tube current of 140 mA. After 6 h of fasting, patients received an intravenous injection of 3 MBq/kg [^18^F]FDG. About 60 min after radiopharmaceutical administration, images were obtained from the skull base to the midthigh. Multimodal image analysis was carried out using a dedicated console (AW Server 4.7, General Electrics, Milwaukee, WI, USA).

Post-treatment scans were reviewed according to Hopkins, Deauville, and Cuneo criteria (Table 1) by two expert nuclear medicine physicians (C.F. and A.R.P.). Observers had access to pre-treatment disease stage and pre-treatment imaging but were blind to patient outcomes and the other reviewer's assignment. A visual score was assigned both to the primary tumor site and lymph nodes. Then, a per-patient final score for each scale was chosen, corresponding to the highest score between the two sites.

**Table 1:** Hopkins, Deauville, and Cuneo criteria for HNSCC post-treatment evaluation

	Score 1	Score 2	Score 3	Score 4	Score 5	Score 6
Hopkins	Minimal uptake < IJV	Minimal uptake > IJV but < liver	Diffuse uptake > IJV and liver	Moderate focal uptake > liver	Intense focal uptake > > liver	
Deauville	No uptake	Minimal uptake < MBP	Low-grade uptake > MBP but < liver	Moderate focal uptake > liver	Intense focal uptake > 2 × liver or new lesions	
Cuneo	No uptake	Residual uptake > MDP but < liver + absent local background	Residual uptake > MDP but < liver + local background	Focal uptake > liver + local background	Focal uptake > liver + absent local background	Focal uptake > > liver

*IJV* internal jugular vein, *MBP* mediastinal blood pool

As usual, scores of 1–3 for Hopkins and Deauville scores and 1–4 for the Cuneo criteria were considered a metabolic response. Further analysis was performed considering the intermedium score 3 for Hopkins and Deauville and score 4 for Cuneo criteria as no response.

To assess the secondary study endpoint, SUV_max_ of the lymph node with the highest [^18^F]FDG uptake on restaging PET/CT scans of both indeterminate and positive cases were collected, if present, together with the corresponding product of the two largest diameters (mm^2^) measured on the co-registered CT images. Accordingly, the delta SUV_max_ (ΔSUV_max_), calculated as the difference between the highest lymph node SUV_max_ at the baseline and the post-treatment PET/CT, was registered.

### Statistical analysis

Post-treatment disease status was determined by any evidence of clinical, pathological, and/or radiological locoregional progression in the first 3 months from the final fraction of radiotherapy treatment. Progression-free survival (PFS) was defined as the time from the last day of treatment to the date of first recurrence (including primary tumor or regional lymph nodes recurrence as well as distant metastasis). Overall survival (OS) was defined as the time from the cancer diagnosis to death from any cause or last follow-up.

Sensitivity, specificity, PPV, NPV, and overall accuracy were calculated for each score. PFS and OS were estimated by the Kaplan–Meier method. In order to discriminate progression *vs* treatment-related changes, the optimal cutoff values of SUV_max_, its delta, and the diameters product of the lymph node with the highest [^18^F]FDG uptake were evaluated using the ROC curve. In addition, to select potential prognostic factors a Cox proportional hazards regression analysis was performed by calculating the hazard ratios (HR) and corresponding 95% confidence intervals (CI 95%). The statistical significance level was set at *p* < 0.05. Statistical analysis was performed using SPSS for Windows software (Version 28.0; IBM Corp., Armonk, New York, USA).

## Results

### Patients’ characteristic

Among 812 [^18^F]FDG PET/CT scans performed in our Nuclear Medicine Department on 329 patients with histologically proven HNSCC, a total of 43 patients (median age 57 y; M:F = 32:11) met the inclusion criteria and were ultimately recruited for the analysis. The most frequent primary tumor site was the oropharynx (19/43, 44.2%), but HPV status was known only in 4 patients. Other primary tumor sites were the nasopharynx in 9/43 (21%) patients, larynx in 8/43 (18.6%), salivary glands in 4/43 (9.3%), and hypopharynx in 1/43 (2.3%), while 2/43 (4.6%) patients present an occult HNSCC.

Most patients experienced a stage IV (39/43, 90.7%), and a minority had a III stage (4/43, 9.3%) at diagnosis. Concurrent CRT was the primary treatment in most patients (93%), followed by RT alone in 3 patients (7%).

The median time between the end of treatment and the restaging [^18^F]FDG PET/CT was 5 months (range 2–11). The median time between diagnosis and the last follow-up was 48 months (range 7–153). In the first three months after the first restaging PET/CT exam, 15/43 (35%) patients showed disease progression in any site. During follow-up 19/43 (44%) patients experimented recurrence or metastatic disease; in particular, 5/19 (26%) experimented a primary tumor site recurrence, 4/19 (21%) had a locoregional lymph node involvement, 4/19 (21%) both primary tumor site and lymph node recurrence and 6/19 (32%) a metastatic disease (5 lung and 1 liver metastasis). Finally, 18/43 (42%) patients died during the follow-up.

Patients’ characteristics were resumed in Table 2.

**Table 2:** Patient’s characteristics

Characteristics	Value (%)
Total patients 43	
*Age* (years), median (range)	57, 15–81
Gender	
Male	32 (74%)
Female	11 (26%)
Primary site	
Oropharynx	19 (44.2%)
Nasopharynx	9 (21%)
Larynx	8 (18.6%)
Salivary glands	4 (9.3%)
Occult	2 (4.6%)
Hypopharynx	1 (2.3%)
Treatment	
Concurrent chemoradiotherapy	40 (93%)
Radiotherapy	3 (7%)
Stage	
III	4 (9%)
IV	39 (91%)

### Visual score performance and survival correlation

The cumulative sensitivity, specificity, PPV, NPV, and accuracy were 87%, 86%, 76%, 92%, and 86% for the Hopkins score, whereas 93%, 79%, 70%, 96%, and 84% for the Deauville score, respectively. Conversely, the Cuneo score reached the highest specificity and PPV (93% and 78%, respectively) but the lowest sensitivity (47%), NPV (76%), and accuracy (77%). Diagnostic performance concerning the primary tumor site, lymph nodes, and overall score are summarized in Table 3.

**Table 3:** Diagnostic performance for each score in relation to the primary tumor site, lymph node, and cumulative one considering as metabolic response score 1,2,3 for Hopkins and Deauville and score 1,2,3,4 for Cuneo scores

SITE	SCORE	Sensitivity (%)	Specificity (%)	PPV (%)	NPV (%)	Accuracy (%)
Primary tumor	Hopkins	67	89	77	83	81
	Deauville	80	82	71	88	81
	Cuneo	40	96	86	75	77
Lymph node	Hopkins	47	93	78	76	77
	Deauville	53	93	80	79	79
	Cuneo	33	96	83	73	74
Cumulative	Hopkins	87	86	76	92	86
	Deauville	93	79	70	96	84
	Cuneo	47	93	78	76	77

*PPV* positive predictive value, *NPV* negative predictive value

Shifting patients with intermedium score for each criterion (score 3 for Hopkins and Deauville, and score 4 for Cuneo) into the no-responder group, the cumulative performance of the three criteria revealed a sensitivity, specificity, PPV, NPV, and accuracy of 93%, 71%, 64%, 95%, and 79% respectively for Hopkins score. Deauville and Cuneo score reached a 100% of sensitivity and NPV, indicating that treatment-related changes could often impact the intermedium score. Instead, specificity, PPV, and accuracy were respectively 64%, 60%, and 77% for the Deauville score and 75%, 68%, and 84% for the Cuneo criteria. The comparison between cumulative diagnostic performance considering different risk groups is shown in Table 4.

**Table 4:** Comparison between cumulative diagnostic performance considering two different risks groups for each score: with or without the indeterminate score 3 for Hopkins and Deauville and score 4 for Cuneo criteria

	Hopkins 1 (4,5 = progression) (%)	Hopkins 2 (3,4,5 = progression) (%)	Deauville 1 (4,5 = progression) (%)	Deauville 2 (3,4,5 = progression) (%)	Cuneo 1 (5,6 = progression) (%)	Cuneo 2 (4,5,6 = progression) (%)
Sensitivity	87	93	93	100	47	100
Specificity	86	71	79	64	93	75
PPV	76	64	70	60	78	68
NPV	92	95	96	100	76	100
Accuracy	86	79	84	77	77	84

*PPV* positive predictive value, *NPV* negative predictive value

**Fig. 1:**
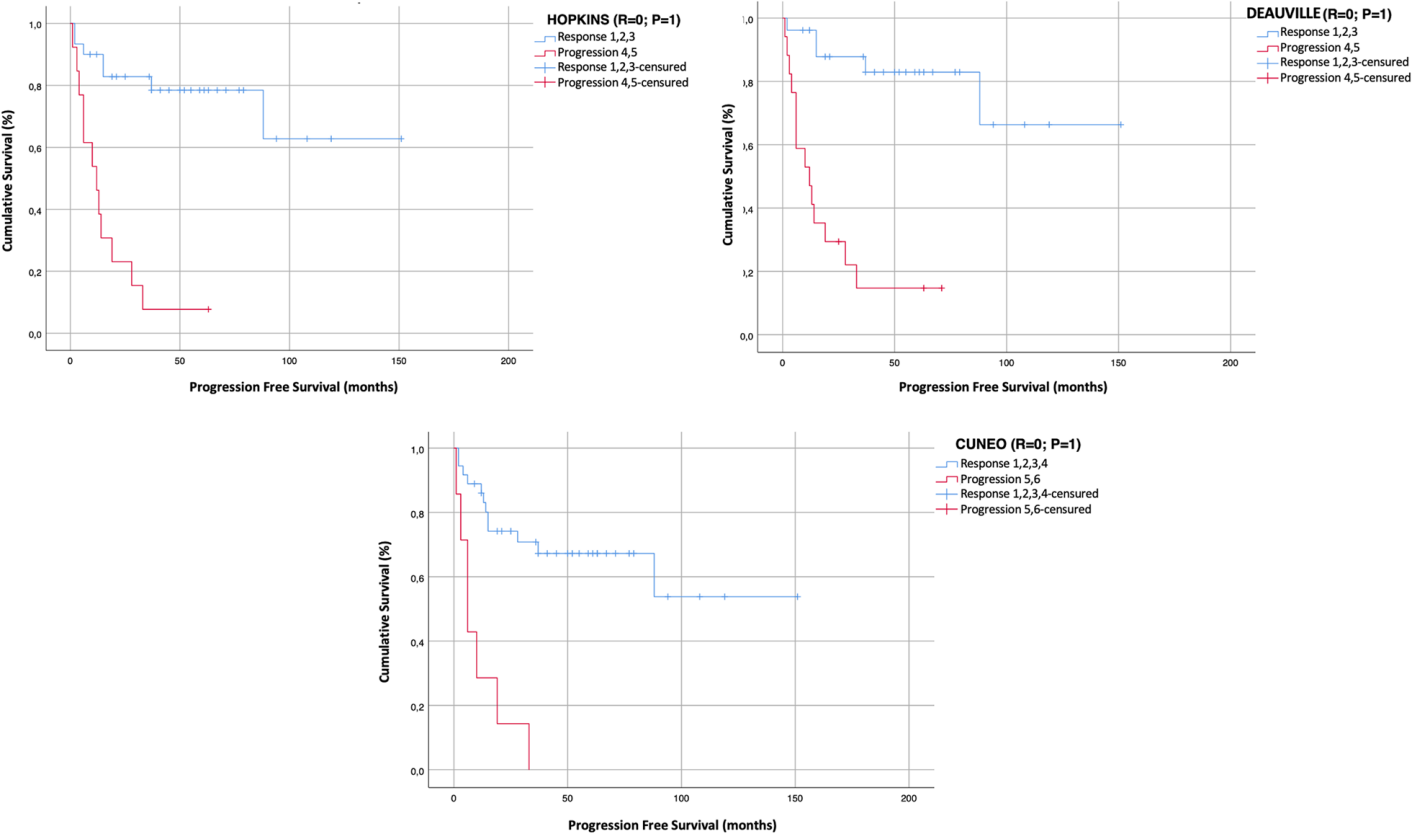
Kaplan–Meier plots show progression free survival of HNSCC patients according to Hopkins, Deauville and Cuneo scores (log-rank Mantle–Cox test, *p* = 0.0001 for all visual scores)

**Fig. 2:**
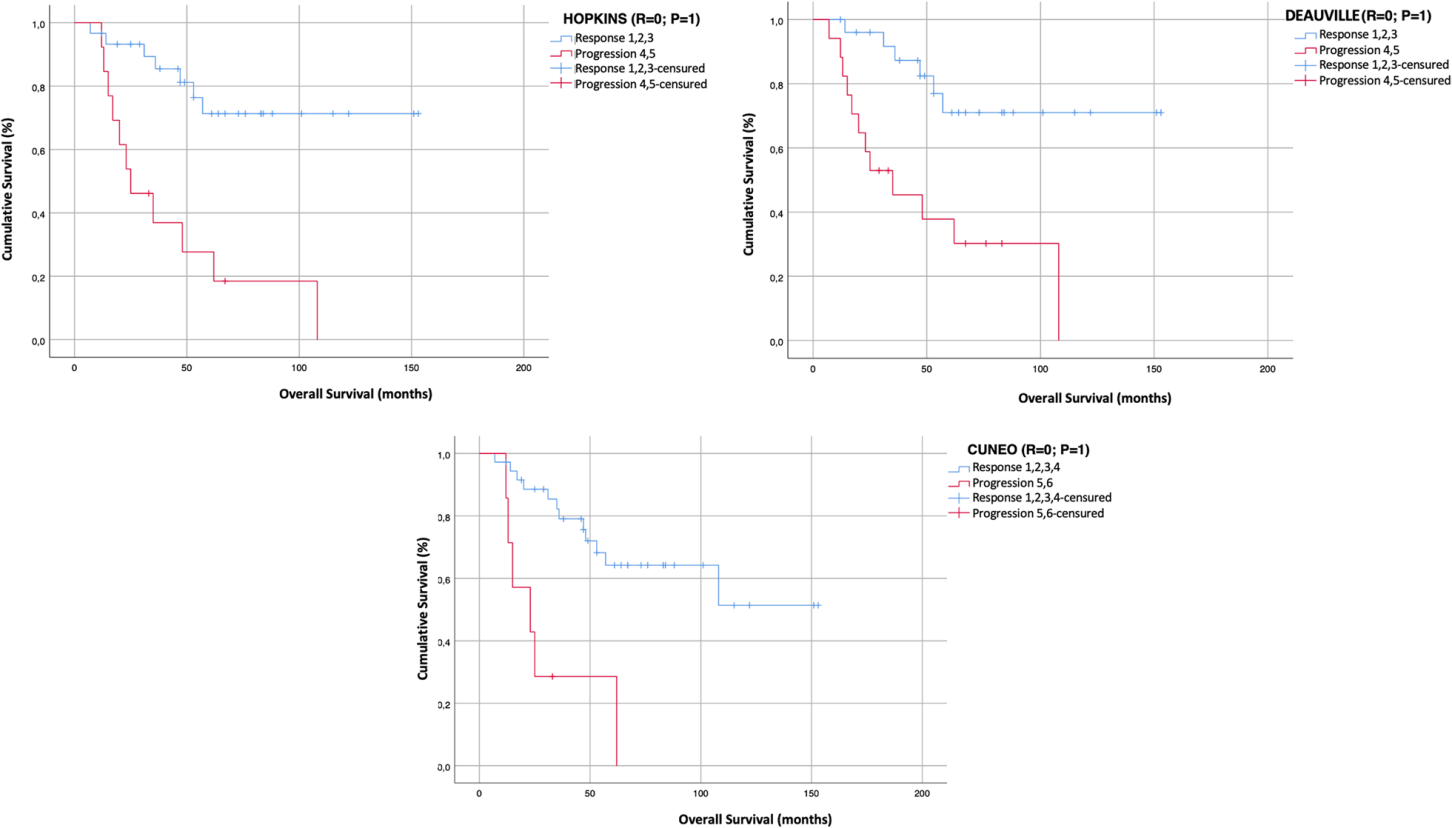
Kaplan–Meier plots show overall survival of HNSCC patients according to Hopkins, Deauville and Cuneo scores (log-rank Mantle–Cox test, Hopkins *p* = 0.05; Deauville *p* = 0.001; Cuneo *p* = 0.002)

Considering the correlation of each visual score with the survival analysis, among the 19 patients who experienced disease recurrence or progression at any site, 16/19 (84%) showed a 4–5 HS, 18/19 (95%) a 4–5 DS, and 17/19 (89%) patients a 5–6 CS. Moreover, among 18 patients who died during follow-up, 12/18 (60%) patients had a 4–5 HS, 15/18 (68%) a 4–5 DS, and 8/18 (64%) a 5–6 CS. Each visual scale significantly correlated with PFS (*p* < 0.0001) and OS (Hopkins *p* = 0.05; Deauville *p* = 0.001; Cuneo *p* = 0.002). Kaplan–Meyer curves are displayed in Figs. 1 and 2.

### Morphological and metabolic lymph node evaluation at restaging PET/CT

Focusing on the restaging [^18^F]FDG PET/CT scans, 19/43 (44.2%) patients showed a residual lymph node uptake. It was categorized as indeterminate in 11/19 using HS (others 8/19 scored 4 or 5), 9/19 with DS (others 10/19 scored 4 or 5), and 5/19 CS (others 14/19 scored 5 or 6).

The mean SUVmax_max_ of residual lymph node uptake was 4.4 (SD ± 2.7), the mean ΔSUV_max_ was − 5.6 (SD ± 7.4) and the mean product of diameters of the lymph node with the highest uptake was 174.8 mm^2^ (SD ± 117.4).

**Fig. 3:**
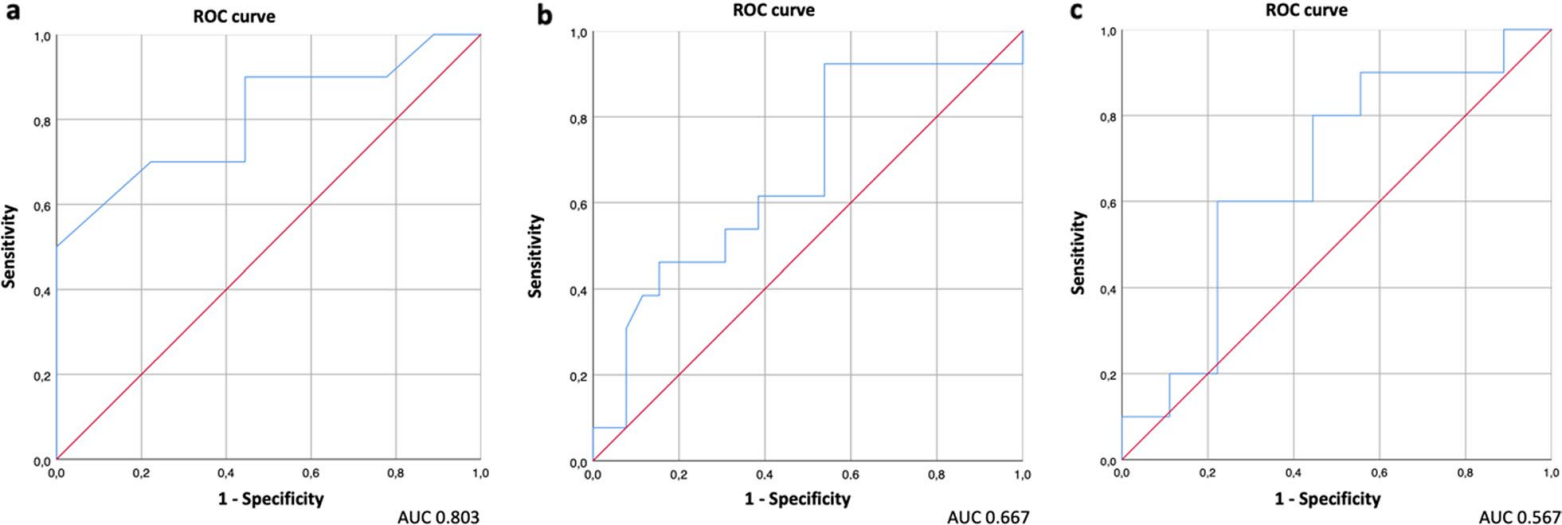
ROC curves and relative AUC of the post-treatment [^18^F]FDG PET SUV_max_ (**a**), the ΔSUV_max_ (**b**), and the product of diameters (**c**) of lymph node with the highest uptake of restaging PET scan

**Fig. 4:**
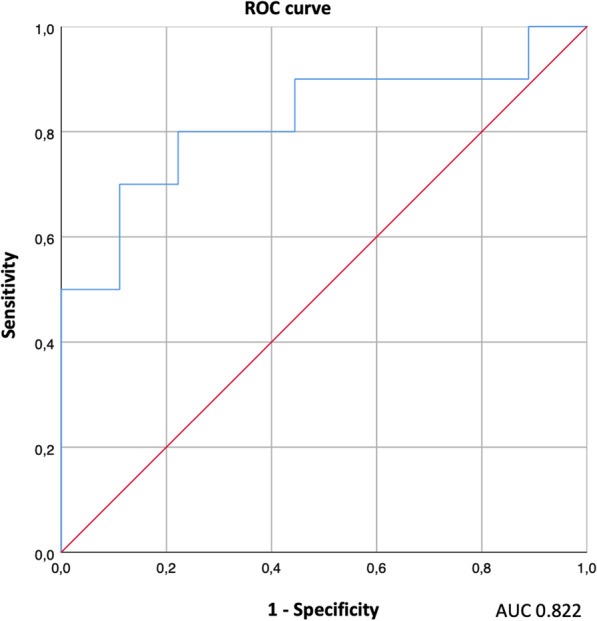
ROC curve and relative AUC of the combination of SUV_max_ and the product of diameters of lymph node with the highest uptake of restaging PET scan

Based on ROC curve analysis results (Fig. 3), a SUV_max_ cut-off of 2.75 extracted from post-treatment [^18^F]FDG PET/CT reached the best area under the curve (AUC) in discriminating progression from treatment-related changes (sensitivity 90%, specificity 56%; AUC = 0.806). The best cutoff for ΔSUV_max_ was − 6.6 (sensitivity 92%, specificity 54%; AUC = 0.667). The ROC curves for the product of diameters did not reach a significative AUC (0.567). The ROC analysis of the combination of the SUV_max_ and the product of diameters (Fig. 4) showed good discriminatory power with AUC of 0.822 (95% CI 0.624–1.0).

In the subgroup of indeterminate and positive cases, the multivariate analysis revealed the Cuneo criteria (HR 0.271, 95% CI 0.082–0.892, *p* = 0.032) and the product of diameters (HR 1.004, 95% CI 1.0–1.009, *p* = 0.051) as prognostic factors for PFS.

## Discussion

Despite the consolidated role of [^18^F]FDG PET/CT in the treatment response evaluation of HNSCC, there is no clear consensus on the optimal interpretation criteria to use in this clinical scenario. Semi-quantitative PET parameters, such as the well-known SUV_max_, have not proved to be reliable indicators probably due to the numerous technical and patient variables that could influence this value, especially in the investigated population (e.g. inflammation and post-treatment effects) [4, 15, 16].

Over the last years, considerable efforts have been made to standardize the reporting system in the surveillance of HNSCC and several qualitative assessment methods for predicting regional disease control have been proposed to find out the best [^18^F]FDG PET visual scale. However, none of them were approved and routinely used up to date. As known, one of the major limits remains the low sensitivity and PPV.

In our study, the head-to-head comparison between scales showed good sensitivity, specificity, and PPV (87%, 86%, and 76% respectively) and a high NPV (92%) of the first introduced Hopkins scale. Comparing our results with the reference one, published by Marcus and colleagues, we reported almost similar specificity and NPV (92.2% and 91.1%, respectively), but a higher sensitivity and PPV compared to 68.1% and 71.1% in the reference study [12].

Similarly, a 2017 study by Kendi et al. [17] on 69 HNSCC patients showed a sensitivity, specificity, PPV, and NPV of the overall therapy assessment of 66.7%, 87.3%, 33%, 96.5% respectively, underlining the higher sensitivity and PPV reached in our study. The diagnostic accuracy of PET/CT response assessment in our study might be affected by the time interval between treatment and follow-up imaging; the later median time point of imaging post-therapy (20 weeks) compared to other studies may explain slightly higher PPV[18].

More recently, other authors proposed to apply the Deauville score, the most famous 5-point standardized Likert scale used for Hodgkin’s and non-Hodgkin’s lymphoma, to post-treatment HNSCC PET assessment [19]. Benjamin et al. conducted a retrospective analysis on 43 HNSCC patients who underwent organ preservation radiotherapy and applied the DS to [^18^F]FDG PET/CT scan. Their study results showed a higher PPV than our (100% *vs* 70%), but only 4/43 patients experienced disease progression (4 or 5 DS) [4].

Another study by Zhong et al., in a larger cohort of 562 patients, compared the diagnostic performance of the Hopkins and Deauville score for the prediction of locoregional control and PFS. The study confirmed the high NPV of both visual criteria, also achieving a very high PPV compared to our as well as to the existing literature (51–78%). However, the authors attributed this higher value to the later median time point of imaging post-therapy (17 weeks) [20].

**Fig. 5:**
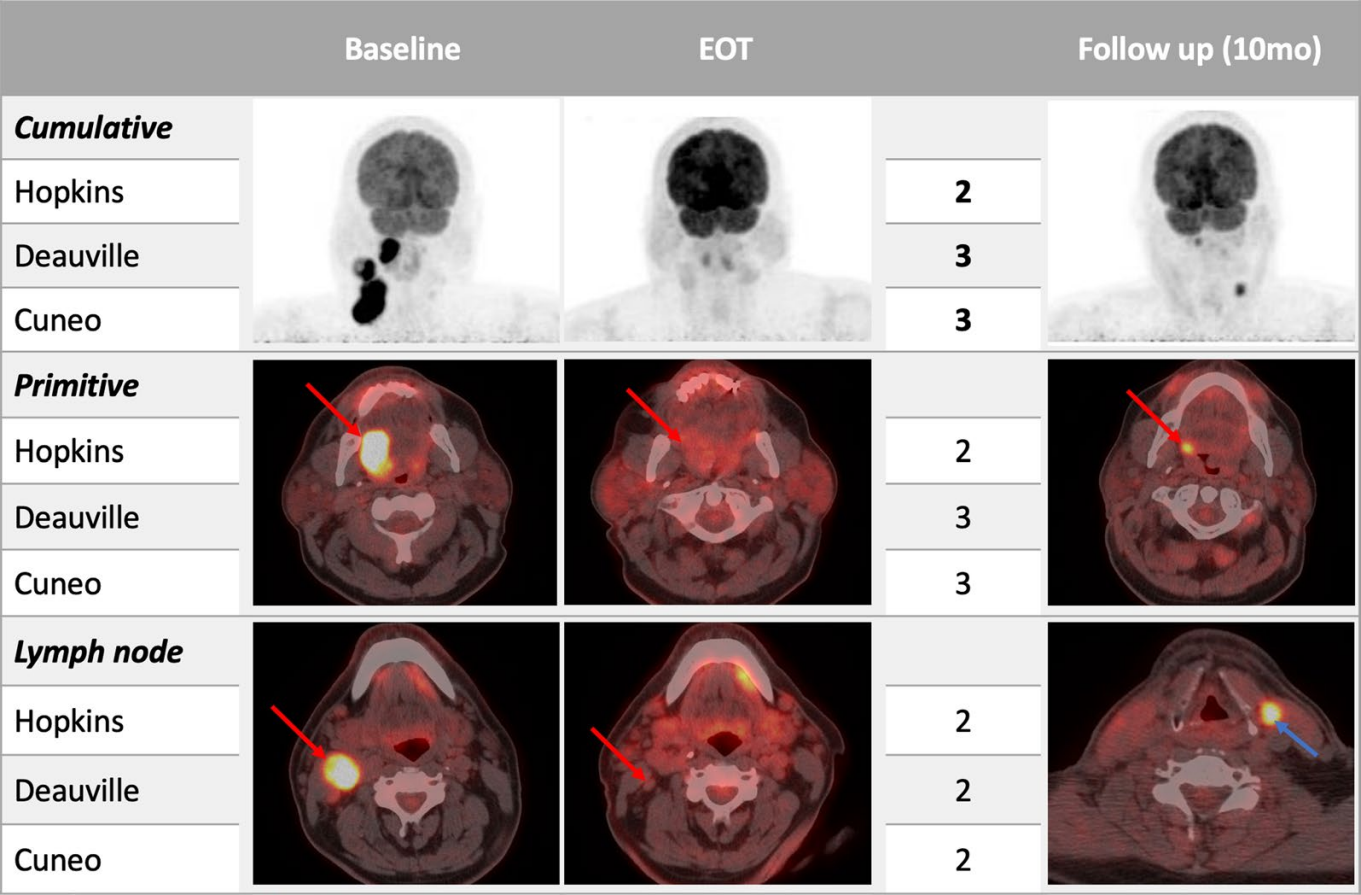
♂, 53yo. with poorly differentiated squamous carcinoma of the posterior tongue histologically proven. Baseline PET/CT showed a pathological FDG-uptake in the primary tumor site as well as in the right cervical and supraclavicular lymph nodes. End-of-treatment (EOT) PET/CT, performed 14 weeks after radiotherapy, showed moderate residual uptake near the primary tumor site first considered as treatment-related and scores undetermined in 2 out of 3 visual scores. However, the follow-up PET/CT, performed about 10 months after EOT, showed a relapse of the disease in the primary tumor site as well as a contralateral lymph node spread

To deal with these conflicting results, in 2020 an Italian Multicentric study introduced a novel 6-point visual system called Cuneo score to find out a higher PPV [14]. The reference article compared the three different PET visual scores, reporting the best PPV for Cuneo score (with scores 3 and 4 clustered with 1 and 2, indicative of the absence of disease) for all categories: 42.9% for the primary tumor, 100% for the nodal involvement, and 50% for the cumulative score. In our cohort, primary tumor, lymph node involvement, and the cumulative Cuneo score reached higher PPV values (86%, 83%, and 78% respectively), at the expense of lower values of sensitivity and NPV, compared to Hopkins and Deauville scores. Our results support the hypothesis proposed by Bonomo et al. of considering 6-point visual criteria to minimize false positive PET findings in post-treatment evaluation of HNSCC, resulting in a better PPV. Further prospective studies with a larger sample are warranted to support the Cuneo criteria. However, since none of the mentioned criteria showed to be able to solve equivocal cases (e.g., Fig. 5) a further repeat PET/CT 3–6 months later is still necessary, as suggested by current guidelines [3].

**Fig. 6:**
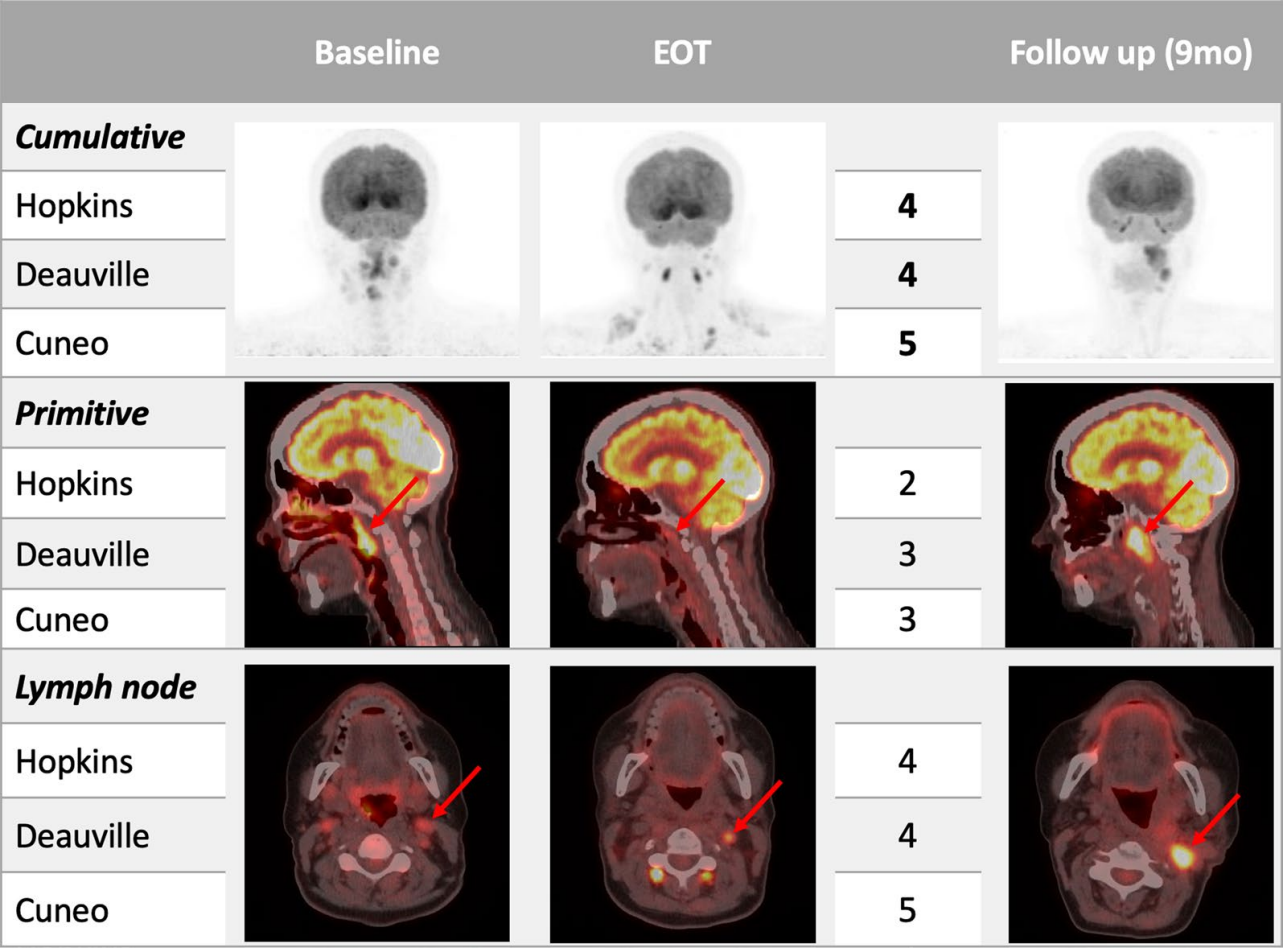
♀, 47yo. with a histological diagnosis of moderately differentiated squamous carcinoma of the oropharynx. Baseline [^18^F]FDG PET/CT showed a high uptake into the oropharynx extended to the nasopharynx with bilateral cervical lymph nodes involvement. Subsequently, the patient underwent concomitant chemo-radiotherapy. End-of-treatment (EOT) PET/CT, performed 17 weeks after radiotherapy, showed mild uptake in the primary site (SUV_max_ 4.1) and a persistent high FDG-uptake in the left cervical lymph node (SUV_max_ 5.8). All visual criteria scored pathologically. The follow-up PET/CT, performed about 9 months after EOT, demonstrated the persistence/recurrence of both locoregional and lymph node disease

**Fig. 7:**
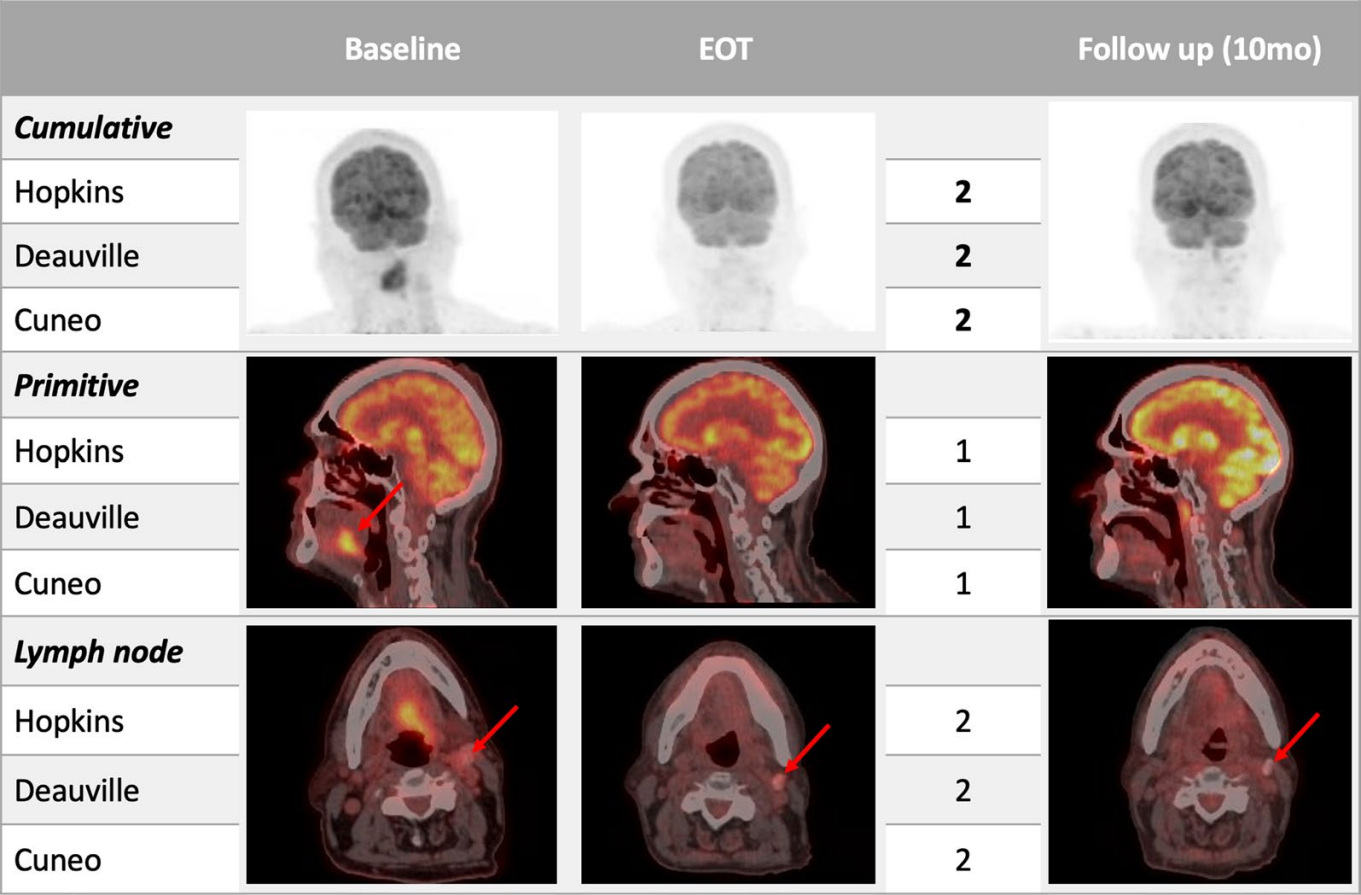
♂, 69yo. with moderately differentiated squamous carcinoma of the oral cavity histologically proven. Baseline PET/CT showed a pathological FDG-uptake in the oral cavity. In addition, a moderate uptake was detected in the left retromandibular lymph node. Afterward, the patient underwent concomitant chemo-radiotherapy. End-of-treatment (EOT) PET/CT, performed 14 weeks after radiotherapy, showed no significant uptake both in the oral cavity and retromandibular lymph node, which appeared also calcific. Scores assigned are consistent with the metabolic response and confirmed at the follow-up PET/CT performed about 10 months later

The survival analysis was consistent with the current literature [21]. All the studied scores again showed their high prognostic value in terms of both PFS and OS, underlining the reliability of a prognostic scoring system in HNSCC patients. Two clinical examples extracted from our sample are shown in Figs. 6 and 7, supporting the prognostic role of each visual score.

Regarding the secondary endpoint of the study, both SUV_max_ extracted from restaging PET and ΔSUV_max_ were able to discriminate lymph node persistent disease from treatment-related changes, showing a good performance in predicting outcomes. However, their cutoff value found (2.75 for SUV_max_, − 6.6 for ΔSUV_max_) needs to be considered with caution, confirming the debated role of semiquantitative analysis in literature.

The added value of our study was the evidence that hybrid imaging, combining morphological and metabolic imaging, could improve [^18^F]FDG PET/CT interpretation in treatment response evaluation. Notably, the ROC analysis of the combination of the SUV_max_ and the product of diameters with the highest uptake on the restaging PET scan showed good discriminatory power with an AUC of 0.822. The multivariate analysis also demonstrated that the Cuneo criteria and the product of lymph node diameters could be considered prognostic factors for PFS. To the best of our knowledge, only one other study from 2016 study attempted to consider lymph node diameters (those with short axis) in the restaging PET scan but failed to assess its usefulness [21]. These results could help the visual interpretation of [^18^F]FDG PET/CT to clarify indeterminate cases and assign new risk classes. The use of contrast-enhanced CT on PET/CT exams could strengthen our hypothesis to better discriminate lymph node involvement [22].

The unknowledge of HPV-status in more than half of our sample limits our study. To note, HPV status is a well-known prognostic factor in HNSCC. HPV-related HNSCCs, which primarily arise in the oropharynx, have a markedly better prognosis compared to HPV-unrelated [15]. One study suggests that lymph node involvement may take longer to regress in patients with HPV-positive disease [23]. Accordingly, we can speculate that the slightly higher PPV obtained in our study than reported rates, could be related to the unknown HPV status as well as the time interval between the restaging PET/CT and the end of radiotherapy. As reported by some literature data, it is possible that HPV-positive patients with equivocal findings at 3-month [^18^F]FDG PET/CT assessment, could avoid a demolitive neck dissection if they undergo a further later PET/CT exam. For positive lymph node disease in HPV-associated patients achieving incomplete response on the 12-week restaging PET/CT, a repeat 16-week PET/CT showed to spare patients from unnecessary surgery [7, 24, 25]. Moreover, the retrospective nature of the study and the relatively small sample size should also be considered.

## Conclusions

In conclusion, each PET-based qualitative therapy assessment was statistically related to prognosis, thus demonstrating the reliability of visual point-scale criteria in HNSCC after definitive non-surgical therapy. The new Cuneo score showed the highest specificity and PPV, but the lowest sensitivity and NPV compared to Hopkins and Deauville criteria. Furthermore, the combination of PET data with morphological features could support the evaluation of equivocal cases.

## Data Availability

The datasets used and/or analyzed during the current study are available from the corresponding author on reasonable request.

## Abbreviations

HNSCC: Head and neck squamous cell carcinoma
[^18^F]FDG PET/CT: Fluorine-18 fluorodeoxyglucose positron emission tomography/computed tomography
SUV: Standardized uptake value
HC: Hopkins criteria
DS: Deauville score
CS: Cuneo score
PFS: Progression-free survival
OS: Overall survival
NPV: Negative predictive value
PPV: Positive predictive value
HPV: Human papillomavirus
